# The Clinical Significance of Pollen and Fungi Concentrations for Allergic Rhinitis: A Three-Year Study

**DOI:** 10.7759/cureus.40397

**Published:** 2023-06-14

**Authors:** Petros Katsimpris, Theodora Deftereou, Gregory Trypsianis, Dimitrios Balatsouras, Gerasimos Danielides, Triantafyllos Alexiadis, Polina Dimitrova, Stergios Lialiaris, Maria Lambropoulou, Michael Katotomichelakis

**Affiliations:** 1 Department of Otorhinolaryngology, Medical School, Democritus University of Thrace, Alexandroupolis, GRC; 2 Laboratory of Histology-Embryology, Medical School, Democritus University of Thrace, Alexandroupolis, GRC; 3 Laboratory of Medical Statistics, Medical School, Democritus University of Thrace, Alexandroupolis, GRC

**Keywords:** mini-rqlq, rqlq, visual analogue scale, total 5 symptoms score, skin prick tests, quality of life, pollen and fungi concentrations, mediterranean region, disease severity, allergic rhinitis

## Abstract

Introduction: The relationship between disease severity and exposure to allergens in allergic rhinitis (AR) patients is not fully clarified presently. We aimed to detect the correlation between airborne pollen and fungi concentrations in a Mediterranean region with symptom scores.

Methods: A total of 98 patients suffering from AR rated their symptoms at the time of exacerbation using the Total 5 Symptoms Score (T5SS) and the Visual Analogue Scale (VAS). Patients’ quality of life (QoL) was estimated by using either disease-specific (Rhinoconjunctivitis Quality of Life Questionnaire (RQLQ) and mini-RQLQ) or generic (Short-Form 36 (SF-36) and Beck Depression Inventory (BDI)) questionnaires. All patients’ responses were correlated with aerobiological data. Skin prick tests (SPTs) were used to detect sensitivities to the most common registered pollen and fungi species.

Results: A significant positive correlation between total pollen and fungi counts and disease-specific questionnaires was found only for the RQLQ. Accordingly, a significant positive correlation was found between total pollen and fungi counts and T5SS (r = 0.655, p = 0.021), with breathing (r = 0.620, p = 0.032) and sneezing (r = 0.660, p = 0.020) being strongly affected. Moreover, a tendency toward a higher VAS score was found as total pollen and fungi counts increased (r = 0.523, p = 0.081).

Conclusion: We found a significant correlation between patients’ symptoms and pollen and fungal air concentrations. Our results emphasize the clinical significance of pollen and fungi maps in everyday clinical practice.

## Introduction

Allergic rhinitis (AR) represents a very common disorder with a prevalence of 20%-30% among adults that rises to 40% in the pediatric population [1,2]. It significantly affects patients’ quality of life (QoL) since it has had a steady upward trend in the last few decades and has led to considerable socioeconomic costs [2]. It is characterized by various clinical manifestations depending on the profile of the sensitized individual, as well as on the aerobiological data of each area [3]. Studies have shown that aeroallergenic patterns differ in various countries as well as in regions within the same country due to geography, climate (winds, temperature, rain, and humidity), vegetation, and cultural factors [3]. Accordingly, their correlation with the clinical manifestations of AR raises an interesting issue. In several reports, it has been found that pollen fluctuations are correlated with the clinical symptoms of respiratory allergies. In particular, some studies highlighted the significant correlation between total pollen and fungi counts and clinical patterns [4-6], while others were unable to prove a similar relationship [7]. Consequently, the relationship between symptoms and exposure to allergens is presently not fully clarified.

In this study, we investigate the clinical importance of the pollen map of a Mediterranean region (Western Thrace/North-East Greece), using records of symptoms from patients who live in the area, and in addition, we observe the correlation between the symptoms and the concentration of allergens. This new information could help clinical practitioners to estimate all the factors that are implicated in AR severity and improve its treatment.

## Materials and methods

Study group

We studied sensitivities to the most frequently registered aeroallergens in the area and symptom scores in a group of 98 allergic rhinitis patients who presented and met all the inclusion criteria, during the years 2015-2017. All patients were resident citizens, at least for the last five years, in the same region. The study was conducted in the Rhinologic Unit of the Department of Otorhinolaryngology at the University Hospital of Alexandroupolis. All patients were evaluated by history, nasal endoscopy, a skin prick test (SPT) with a panel of common aeroallergens, and sinus computed tomography scanning, in order to exclude patients suffering from a disease other than allergic rhinitis. All patients had no abnormal findings in the CT scan. Symptoms were evaluated at the peak of severity, according to their history and clinical examination. Only patients who fulfilled the criteria for AR according to the Allergic Rhinitis and its Impact on Asthma (ARIA) guidelines [8] and were sensitized to pollen and fungi species were involved. Patients with a history of previous nasal surgeries, chronic rhinosinusitis, nasal polyps, malignancy, or a history of urticaria or angioedema were excluded. We also excluded patients who received immunotherapy during the last five years, used nasal or oral corticosteroids less than one month prior to inclusion, or took oral H1 antihistamines less than seven days prior to inclusion. Patients were treated for three months with capsules of 20 mg bilastine once daily per os and nasal spray (azelastine hydrochloride and fluticasone propionate) once into each nostril two times per day, and symptoms were re-evaluated after treatment.

Symptoms and patients’ QoL evaluation

The Total 5 Symptoms Score (T5SS) and the Visual Analogue Scale (VAS) were used in order to evaluate patients’ symptoms. T5SS includes the symptoms of nasal congestion, nasal discharge, sneezing, and itchy noses and eyes. All symptoms were graded from 0 (absent) to 3 (very troublesome), with total scores ranging from 0 to 15. The VAS was assessed on a straight 100 mm line. The patients were asked to mark a point on the line where the left end of the scale symbolized “no symptoms” (0 mm) and the right symbolized the “most severe symptoms” (100 mm). We instructed the patients to mark on the line the severity of the symptoms that they felt at that moment.

The effect of AR symptoms on patients’ QoL was measured either by disease-specific (Rhinoconjunctivitis Quality of Life Questionnaire (RQLQ) and mini-RQLQ) or generic (Short-Form 36 (SF-36) and Beck Depression Inventory (BDI)) questionnaires. The Rhinoconjunctivitis Quality of Life Questionnaire (RQLQ) is a self-reporting questionnaire that measures the everyday problems of patients suffering from rhinoconjunctivitis. It consists of 28 questions and starts with three patient-specific activity questions. We asked our patients to score their problems during the past week and answer each question on a 7-point scale (6 = severe impairment, 0 = no impairment) [9,10]. As far as the mini-RQLQ is concerned, from the 28 items of the RQLQ, only the highest-scoring items were selected (14 questions). It was also developed to detect the effects of rhinoconjunctivitis symptoms on patients’ QoL [10,11]. The SF-36 is a self-reported health assessment questionnaire. It consists of 36 questions that assess eight health parameters: i) physical activity limitations due to health problems (PF); ii) social activity limitations due to physical or emotional issues (SF); iii) role activity limitations because of physical health problems (RP); iv) bodily pain (BP); v) general mental health (well-being and psychological distress) (MH); vi) role activities limitations due to emotional problems (RE); vii) vitality (VT), and viii) general health perceptions (GH) [12]. Finally, the BDI is a 21-item, self-reporting questionnaire that measures depressive symptoms over the past two weeks. The BDI is a popular instrument for quantifying the severity of depressive symptoms and has high internal consistency and reliability. Scores are generally classified as follows: 0-13, minimal or no depression; 14-19, mild depression; 20-28, moderate depression; and 29-63, severe depression [13]. Olfactory function was measured using the Sniffin’ Sticks test package (Burghardt, Wedel, Germany), and patients were divided into normosmics, hyposmics, and anosmics according to the normative values provided for the Mediterranean population [14].

SPT sensitivities

SPTs were performed and evaluated according to the European Academy of Allergy and Clinical Immunology guidelines [15]. The allergen panel (SUBLIVAC; HAL Allergy BV, Leiden, The Netherlands) that we used included pollen and fungi aeroallergens endemic to our region that could cause allergenic symptoms [16]. Specifically, we included in the panel the following allergens: *Dermatophagoides* mix, grasses mix, olive, *Platanus*, spring trees, weeds mix, *Parietaria*, *Alternaria*, *Aspergillus*, *Cladosporium*, and cat and dog danders. The study protocol was approved by the Scientific Council of the University Hospital of Alexandroupolis (ΕΣ/Θ5/6-4-2015). An informed consent form was signed by all participants. The study was conducted in accordance with the Declaration of Helsinki.

Pollen calendar

In parallel, during these years (2015-2017), we measured the atmospheric pollen and fungal content [17]. We collected the samples for the study with the use of a seven-day recording volumetric trap (Burkard Scientific Ltd., Uxbridge, UK), which is a standard equipment for aerobiologic sampling worldwide [18]. The trap was placed on the roof of our academic hospital at 20 m above the ground, according to the manufacturer’s guidelines [18]. The Burkard spore trap collected airborne particles at a rate of 10 L of air per minute continuously for seven days without any attention. A strip of silicone-coated Melinex tape (Burkard) was exposed to air for trapping the spores and was replaced once a week. After that, the tape was cut into 48 mm segments, representing 24-hour periods, in the Histology Lab of the university. These segments were mounted on microscopic slides using Gelvatol mixed with stain (acid fuchsin) to enable visualization under a high-resolution light microscope (Olympus BX40, Olympus Corporation, Tokyo, Japan) at x400 magnification. Pollen grains and fungi spore counts were expressed as pollen grains and fungi spores per cubic meter of air and total pollen and fungi counts. The pollen and fungi calendar was created according to the instructions of the British Aerobiology Federation [19]. Aeroallergens measured were most often described in AR, not only in the Mediterranean and European regions [3,20-23] but also worldwide [24,25]. We counted grasses (family (f.) *Poaceae*/*Gramineae*), trees (f. *Oleaceae*/olive, f. *Cupressaceae*/cypress, f. *Pinaceae*/pine, f. *Betulaceae*/birch, f. *Platanaceae*/plane), weeds (f. *Chenopodiaceae*), and fungi (*Alternaria* spp and *Cladosporium* spp). A specific scale was used for the contraction of the pollen graph, with each level corresponding to a particular amount of pollen grains and fungi spores. The levels embraced a total sum of pollen grains per 10 days as follows: first level: 1 to 2; second level: 3 to 5; third level: 6 to 11; fourth level: 12 to 24; fifth level: 25 to 49; sixth level: 50 to 99; seventh level: 100 to 199; eighth level: 200 to 399; ninth level: 400 to 799; 10th level: 800 to 1,599; and 11th level: a sum of more than 1,600 pollen grains per 10 days. Thus, we obtained a decrease in the interaction between external factors and pollen concentrations [23].

Statistical analysis was performed using SPSS version 19.0 (IBM, Armonk, NY, USA). All quantitative variables were expressed as the mean ± standard deviation (SD), whereas qualitative variables were expressed as frequencies (and percentages, %). The normality of quantitative variables was confirmed with the Kolmogorov-Smirnov test. The correlation between patients’ characteristics and total pollen counts was examined using Pearson’s correlation coefficient. All tests were two-tailed, and statistical significance was accepted at p values <0.05.

## Results

Patient characteristics

Ninety-eight AR patients were studied (mean age: 31.66 ± 15.23 years; range: 9 to 79 years; median: 31 years); 40 (40.8%) were males with a mean age of 28.70 ± 16.14 years, and 58 (59.2%) were females with a mean age of 33.71 ± 14.35 years. They all suffered from AR, whereas 11 (11.2%) had asthma and 60 (61.2%) were hyposmiacs (Threshold Discrimination Identification (TDI) score: 16-34.5). All patients, according to their occupations, were chronically exposed outdoors to various allergens. According to the SPTs, 17 patients (17.4%) were mono- and 81 patients (82.6%) were poly-sensitized. In total, patients were found to be sensitized mostly to *Dermatophagoides* (77.6%), grasses (61.2%), olives (45.9%), and *Alternaria* (30.6%). Other less common sensitivities were detected in cat dander (23.5%), spring trees (20.4%), and *Parietaria* (19.4%). All patients’ characteristics and sensitivities are presented in Table 1.

**Table 1:** Patients’ characteristics TDI: Threshold Discrimination Identification.

	Patients	Percent (%)
Gender		
Males	40	40.8
Females	58	59.2
Age		
≤20 years	29	29.6
21-40 years	40	40.8
>40 years	29	29.6
Smoking history	30	30.6
Family history	43	43.9
Asthma history	11	11.2
Seasonality		
Intermittent	32	32.7
Persistent	66	67.3
Sensitivity to allergens		
1	17	17.3
2	25	25.5
>2	56	57.1
Olfaction (TDI score)		
Hyposmia	60	61.2
Normosmia	38	38.8
Patients’ sensitivity to allergens		
Grasses (*Lolium perenne*, Ryegrass; *Poa pratensis*, Kentucky blue; *Phleum pratense, *Timothy)	60	61.2
Οlive	45	45.9
Dermatophagoides	76	77.6
Alternaria	30	30.6
Cat dander	23	23.5
Weeds (*Urtica dioica*, Stinging nettle; *Plantago lanceolata*, English plantain; *Artemisia vulgaris, *Mugwort; *Rumex acetosella*, Red sorrel)	8	8.2
Platanus	1	1.0
Aspergillus	10	10.2
Spring trees (*Corylus avellana*, Hazel, *Betula verrucosa*, Birch, *Alnus glutinosa, *Alder)	20	20.4
Parietaria	19	19.4
Dog dander	8	8.2
Cladosporium	3	3.1

Correlations between patients’ symptoms and pollen and fungi counts

The correlation between patients’ symptoms and pollen and fungi counts is shown in Table 2. 

**Table 2:** Symptom correlations to allergen concentrations expressed as Pearson’s r correlation coefficient VAS: Visual Analogue Scale; BDI: Beck Depression Inventory; RQLQ: Rhinoconjunctivitis Quality of Life Questionnaire; SF-36: Short-Form 36; T5SS: Total 5 Symptoms Score.

	Total	
	R	p value
VAS	0.523	0.081
BDI	0.416	0.178
RQLQ		
Sleep	-0.516	0.086
Non-hay (non-eye, non-nasal) symptoms	0.061	0.852
Practical problem	0.585	0.046
Nasal symptoms	0.545	0.067
Eye symptoms	0.538	0.071
Activity	0.635	0.026
Emotion	-0.593	0.042
Total	0.594	0.042
Mini-RQLQ		
Activity limitations	0.556	0.060
Practical problems	0.594	0.042
Nose symptoms	0.409	0.186
Eye symptoms	0.432	0.162
Other symptoms	-0.381	0.222
Total	0.481	0.114
SF-36		
PF (physical function)	-0.281	0.376
SF (social function)	0.402	0.195
RP (role physical)	-0.688	0.013
RE (role emotional)	-0.250	0.434
MH (mental health)	-0.549	0.064
VT (vitality)	0.381	0.222
BP (bodily pain)	-0.096	0.766
GH (general health)	-0.103	0.749
Total	-0.317	0.316
T5SS		
Difficulty breathing	0.620	0.032
Sneezing	0.660	0.020
Rhinorrhea	0.535	0.073
Eye symptoms	0.521	0.082
Nasal pruritus	0.423	0.170
Total	0.655	0.021

**Figure 1:**
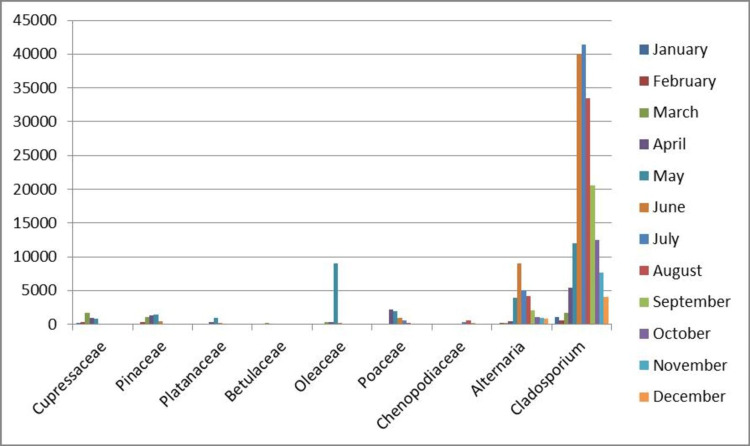
Pollen averages (2015-2017 years), expressed in total pollen grains and fungi spores

The three-year pollen and fungi calendar (2015-2017) is presented in Figure 1 and was expressed as total pollen grains and fungi spores [17].

Total pollen and fungi counts in the atmosphere were strongly positively correlated with the RQLQ total score (r = 0.594, p = 0.042). Regarding the specific symptoms of RQLQ, total pollen counts were positively correlated with a practical problem (r = 0.585, p = 0.046) and activity (r = 0.635, p = 0.026) and negatively correlated with emotion (r = -0.593, p = 0.042); correlations of marginal significance were observed with sleep (r = -0.516, p = 0.086), nasal (r = 0.545, p = 0.067), and eye symptoms (r = 0.538, p = 0.071). Among the specific symptoms of mini-RQLQ, practical problems were significantly correlated with total pollen and fungi counts (r = 0.594, p = 0.042), while the positive correlation of activity limitations with total pollen counts (r = 0.556, p = 0.060) was of marginal significance. Of the domains of SF-36, physical role (PR) (r = -0.688, p = 0.013) and mental health (MH) (r = -0.549, p = 0.064) indicated negative correlations with total pollen and fungi counts. A strong positive significant correlation was also observed between total aeroallergens and T5SS (r = 0.655, p = 0.021). In more detail, total pollen and fungi counts were positively correlated with difficulty in breathing (r = 0.620, p = 0.032), sneezing (r = 0.660, p = 0.020), runny nose (r = 0.535, p = 0.073), and eye symptoms (r = 0.521, p = 0.082), although the last two correlations were of marginal significance. Finally, a tendency toward higher VAS score was found as total aeroallergens increased (r = 0.523, p = 0.081). Changes in RQLQ and T5SS after treatment in relation to sensitivities to aeroallergens are shown in Table 3.

**Table 3:** Change of RQLQ and T5SS in relation to sensitivities to aeroallergens RQLQ: Rhinoconjunctivitis Quality of Life Questionnaire; T5SS: Total 5 Symptoms Score.

	RQLQ change (Mean ± SD)	p value	T5SS change (Mean ± SD)	p value
Grasses		0.031		0.037
No	-0.80 ± 0.47		-4.84 ± 1.42	
Yes	-1.06 ± 0.63		-5.53 ± 1.66	
Οlive		0.524		0.030
No	-0.99 ± 0.68		-4.94 ± 1.78	
Yes	-0.92 ± 0.46		-5.64 ± 1.28	
Dermatophagoides		0.535		0.653
No	-1.03 ± 0.78		-5.41 ± 2.02	
Yes	-0.94 ± 0.53		-5.22 ± 1.48	
Alternaria		0.963		0.583
No	-0.96 ± 0.63		-5.21 ± 1.64	
Yes	-0.95 ± 0.49		-5.40 ± 1.54	
Cat danders		0.669		0.469
No	-0.94 ± 0.61		-5.20 ± 1.60	
Yes	-1.00 ± 0.53		-5.48 ± 1.62	
Weeds (*Chenopodium*)		0.124		0.101
No	-0.98 ± 0.60		-5.34 ± 1.61	
Yes	-0.65 ± 0.31		-4.38 ± 1.30	
Αspergillus		0.345		0.781
No	-0.94 ± 0.60		-5.25 ± 1.61	
Yes	-1.13 ± 0.52		-5.40 ± 1.58	
Spring trees		0.071		0.047
No	-0.90 ± 0.50		-5.10 ± 1.49	
Yes	-1.17 ± 0.84		-5.90 ± 1.89	
Parietaria		0.842		0.154
No	-0.95 ± 0.61		-5.15 ± 1.59	
Yes	-0.98 ± 0.49		-5.74 ± 1.59	
Dog danders		0.041		0.264
No	-0.92 ± 0.59		-5.21 ± 1.60	
Yes	-1.36 ± 0.34		-5.88 ± 1.55	
Cladosporium		0.053		0.662
No	-0.98 ± 0.59		-5.25 ± 1.61	
Yes	-0.31 ± 0.26		-5.67 ± 1.53	

Higher improvement of RQLQ values was associated with sensitivity to grasses (p = 0.031) and dog epithelia (p = 0.041), while higher improvement of T5SS was associated with sensitivity to grasses (p = 0.037), olives (p = 0.030), and spring trees (p = 0.047).

## Discussion

It is widely accepted that there is a correlation between the geographical and climate conditions of an area, the pollen and fungal concentrations, and the clinical manifestations of respiratory allergies via sensitization and response pathways [3,26]. However, it is of great significance to know whether there is a correlation between the concentrations of pollen and fungi in the air and patients’ symptoms, and if so, which are the best clinical tools to use in order to detect this correlation. 

Accordingly, we first explored the sensitization prevalence to pollens and fungi in allergic rhinitis patients, who lived in the Western Thrace/NE Greece region for more than five years. At the same time, the pollen and fungi map of the region was constructed. The first thing that we clearly observed was the finding that there is no correlation between the SPT sensitization prevalence and pollen grain and fungal spore concentrations in the air. This finding is in total agreement with our previous work [5,6]; however, the duration of the measurements in this study was longer, and as a result, the conclusions are more safe. Therefore, it is important to mention that most of the patients (61.2%) were sensitized to grasses, although f. *Poaceae* species accounted for 21% of the total pollen. This discrepancy can be attributed to the high allergenicity of *Poaceae* species [+++] [27]. The same was found for olives, which accounted for 45.9% of SPT sensitivities, whereas the pollen grains in the air represented 35.7% of the total number of pollens. On the other hand, spring trees were detected to be responsible for 10.2% of the sensitivities, although their concentrations in the atmosphere were much larger (38.3%). This can be attributed to their low allergenicity [+] [27]. D’Amato and Lobefalo [28] showed that skin sensitization to different pollens is changing between areas in the Mediterranean region, due to differences in the environmental characteristics of a region.

Additionally, another clinically important finding was the percentage of AR patients (57.1%) who were polysensitized (to more than two pollens). This fact in combination with our previous finding [17] that most aeroallergens are presented in clinically significant concentrations throughout the year emphasizes the need to detect which clinical parameters and tools are to be estimated in order to detect the early clinical appearance of AR. According to previous studies, concentrations of 20 pollen grains/m^3^ in the atmosphere are usually sufficient for respiratory allergy symptoms to be present in sensitized individuals [29].

In order to detect the association of the pollen and fungi concentrations in the atmosphere with clinical symptoms and how patients are affected, we used clinical parameters as expressed by the T5SS and the VAS, and questionnaires for QoL assessment, either disease-specific or generic ones. As concerns clinical symptoms only the T5SS was found to be significantly correlated to aeroallergen counts. In particular, total pollen and fungi counts were strongly correlated with T5SS (r = 0.655, p = 0.021), and additionally, difficulty breathing (r = 0.620, p = 0.032) and sneezing (r = 0.660, p = 0.020) were the most troublesome symptoms that affected patients during the main pollination period. Other symptoms such as runny nose (r = 0.535, p = 0.073), itchy nose ( r= 0.423, p = 0.170), and ocular involvement (r = 0.521, p = 0.082) showed a weaker relationship with marginal significance. As far as VAS was concerned, a tendency for statistically significant correlation (r = 0.523, p = 0.081) was found between the clinical conditions represented in the scale and total aeroallergen fluctuations. The prevalence of nasal symptoms is confirmed in other publications, as well [4,30].

Regarding the question of how allergy symptoms affect patients’ QoL, we used two generic (SF-36, BDI) and two disease-specific (RQLQ, mini-RQLQ) questionnaires. It is important to mention that a strong positive and significant correlation between total pollen and fungi counts in the atmosphere and patients’ QoL was detected only for the RQLQ total score (r = 0.594, p = 0.042), whereas for the mini-RQLQ and pollen and fungi counts, there was no significance. Hence RQLQ presents a higher level of trust in its reliability when it comes to describing allergic rhinitis compared to its short version. Regarding their items, we found that total aeroallergen counts were positively correlated with questions referring to practical problems (r = 0.585, p = 0.046) and activity (r = 0.635, p = 0.026) and negatively correlated with emotion (r = -0.593, p = 0.042); correlations of marginal significance were observed with sleep (r = -0.516, p = 0.086), nasal (r = 0.545, p = 0.067), and eye symptoms (r = 0.538, p = 0.071). Among the symptoms of mini-RQLQ, a significant correlation with total pollen and fungi counts was only found for practical problems (r = 0.594, p = 0.042), whereas the positive correlation of activity limitations with total pollen and fungi counts (r = 0.556, p = 0.060) was of marginal significance. Finally, the Beck Depression Inventory (BDI), that refers to the phycological profile of the individuals and the 36-item Short-Form Survey (SF-36), which describes the general quality of life, did not show any strong difference equivalent to pollen and fungi loads throughout the year, as they are dependent on a variety of factors. Of the domains of the SF-36, physical role (r = -0.688, p = 0.013) and mental health (r = -0.549, p = 0.064) indicated negative correlations with total pollen and fungi counts. As a result, we conclude that the more specific questionnaires exhibit a stronger correlation between aeroallergens and clinical patterns and, accordingly, should be preferred when evaluating AR sufferers. 

Finally, as concerns treatment results, we found that patients who were sensitive to grasses presented significant improvement in their QoL after treatment compared to other allergens. Similarly, the T5SS was significantly improved in patients who were sensitive to grasses, olive, and spring trees after three-month treatment with oral anti-histamines and nasal sprays of corticosteroids. This can also be explained by the fact that pollen concentrations probably could be decreased after three months of treatment, so that it might additionally contribute to the decreased symptom scores. A limitation of this study was that our sample was rather small and multiple variables were analyzed. However, our previous study on a similar topic conducted in the same setting corroborates our present findings, since we found similar results.

## Conclusions

In the present study, we found a significant correlation between allergen loads and symptoms. Higher total pollen and fungi counts signify more severe symptoms, specifically more difficulty breathing and sneezing. In patients’ QoL, these symptoms were correlated with more severe practical and activity problems and negatively correlated with emotion. Our findings highlight the importance of the application of pollen and fungi maps in everyday clinical practice. Moreover, as concerns treatment results, we found that patients who were sensitive to grasses presented significant improvement in their QoL after treatment compared to other allergens and similarly the T5SS was significantly improved in patients who were sensitive to grasses, olive, and spring trees after three-month treatment with a combination of anti-histamines and nasal corticosteroids. As concerns the questionnaires for QoL evaluation, it is important to mention that, according to our findings, the disease-specific ones are preferable to the generic ones. Although further research in this field is needed, our findings add new knowledge that could possibly further improve the diagnostic, preventive, and therapeutic techniques for allergic rhinitis.
